# Breast contracture and skin sclerosis following 20 years of polyacrylamide hydrogel migration in a patient with familial vitiligo: a case report

**DOI:** 10.1186/s12893-021-01097-3

**Published:** 2021-02-26

**Authors:** Bin Wang, Jiaming Sun, Jing Tong

**Affiliations:** Department of Plastic Surgery, Union Hospital, Tongji Medical College, Huazhong University of Science and Technology, Wuhan, 430022 China

**Keywords:** Polyacrylamide hydrogel injection, Breast augmentation, Breast contracture

## Abstract

**Background:**

Breast augmentation with polyacrylamide gel (PAAG) injection was approved in China in 1998 and later banned in 2006. The ban ensued numerous complaints from patients such as pain, induration, deformation, infection, displacement, and milk deposition associated with PAAG injection. To date, no study has investigated the long-term effect of PAAG migration on autoimmune diseases.

**Case presentation:**

We report a rare case of a 49-year-old female patient with familial vitiligo who receiving PAAG injection for breast augmentation. The patient reported to have felt persistent movement of PAAG in her thoracoabdominal area for almost 20 years. Furthermore, the PAAG-induced chronic inflammation that aggravated vitiligo, which in turn promoted skin sclerosis. This damaged the breast contracture, increased chest tightness and induced mild breathing problems.

**Conclusion:**

Here, we present a rare case in which a patient with a family history of vitiligo experienced long-term complications after receiving PAAG injection for breast augmentation. This case highlights the relationship between vitiligo, migration of PAAG and tissue hardening and skin contraction.

*Level of evidence*: Level V

## Background

Polyacrylamide gel (PAAG), a hydrophilic biomaterial comprising 2.5% cross-linked polyacrylamide and 97.5% water, was first manufactured in Ukraine in the late 1980s. In China, it was approved for use in breast augmentation in 1998. However, it usage has been associated with several complications in most patients [1] and hence banned by the Chinese State Food and Drug Administration in April 30, 2006 [2–4]. PAAG injection Given that thousands of women received the PAAG injection, it is important to investigate the outcomes of breast augmentation with PAAG to better understand the full landscape of its complications and design effective interventions. Some of the reported complications include contour abnormalities, abnormal skin sensation, pain, induration, malignant breast tumors, aseptic inflammation, leakage, and hematoma [5]. To date, no study has investigated the relationship between the long-term outcomes of PAAG injection for breast augmentation and vitiligo.

This study reports a rare, unresolved, and challenging case of extensive tissue hardening and skin sclerosis accompanied by PAAG gel movement in the thoracoabdominal area, which resulted in white patches as a result of vitiligo that runs in the patient’s family.

## Case representation

**Fig.1:**
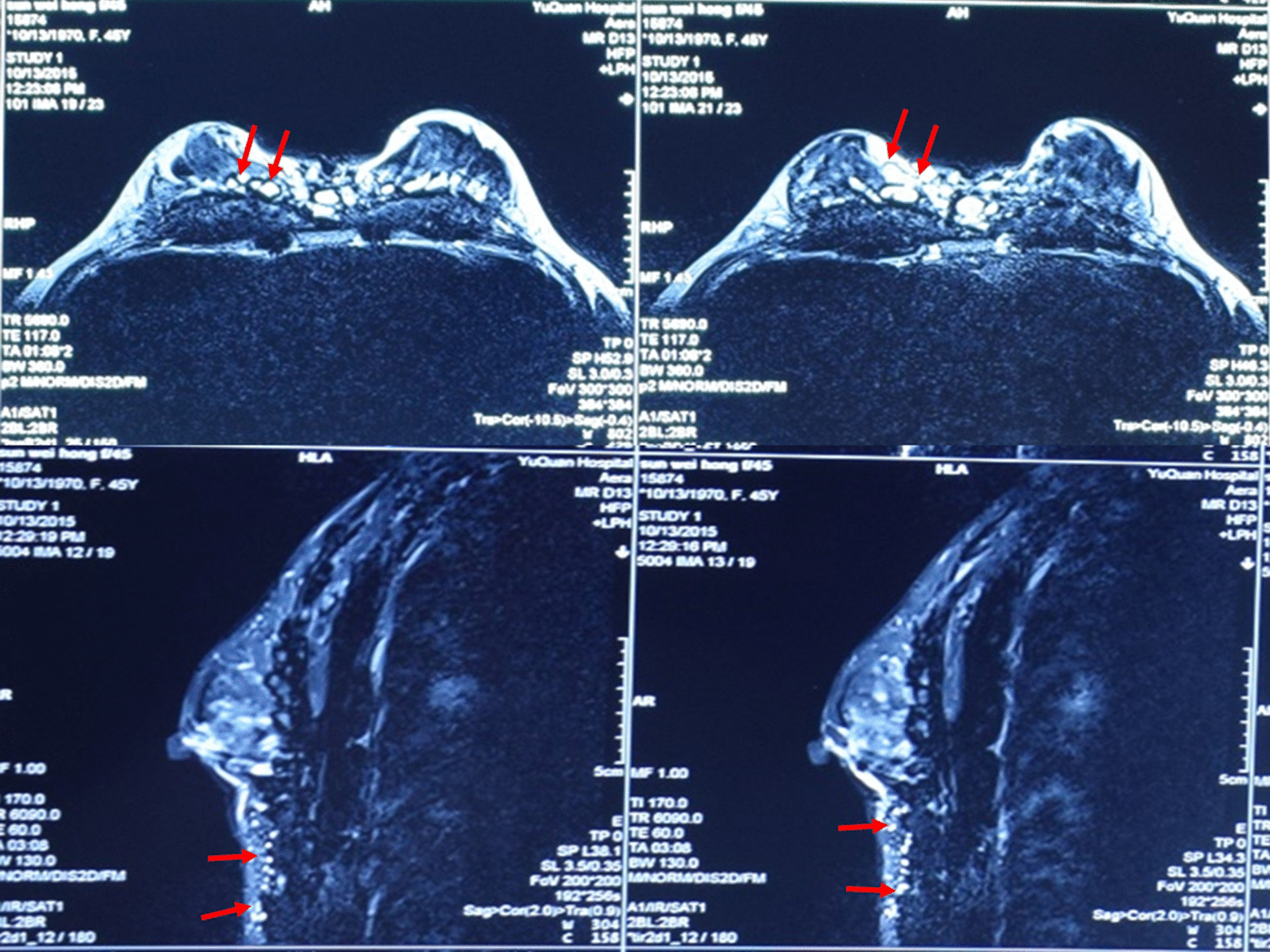
Bilateral breast MR images. (upper) injected PAAG appears bright in the glandular space on T2-weighted image, PAAG in the subcutaneous and mammary gland (arrow); (lower) PAAG in subcutaneous tissues of the chest and abdomen (arrow)

The reported case involves a 49-year-old female patient who had experienced chest tightness for one year. She had untreated familial vitiligo since childhood, and received one-time PAAG injection for breast augmentation 20 years ago. The dosage and the initial injection plane were unknown. Except familial vitiligo, the patient had no other significant past medical history or family history of cancer. And the patient did not smoke or drink alcohol. A few months after the operation, the PAAG began to spread to abdominal regions. Specifically, it was detected in the infraclavicular, subcutaneous tissue of right thoracic wall, perineum, and left side of the upper back. In addition, the skin and soft tissues adjacent to the areas invaded by PAAG became harder and formed numerous hard lumps, especially in the breasts. However, she reported no fever, pain or discomfort. Due to shame and fear, she did not seek medical advice for 15 years. In 2015, she learnt through the Internet of the dangers associated with PAAG and sought medical advice from a physician in November, 2015. She was then hospitalized in the Chinese Academy of Medical Sciences Plastic Surgery Hospital. Chest and abdomen MRI T2-weighted sequence images showed the presence of PAAG gel in many places, including the chest and abdomen wall (Fig. 1). In the chest, it was present in subcutaneous tissue and breast gland. Needle biopsy requested by her excluded the possibility of malignant lesions, scleroderma or granuloma. The patient refused to be operated because of high surgical cost, and complications, especially delayed wound healing.

**Fig.2:**
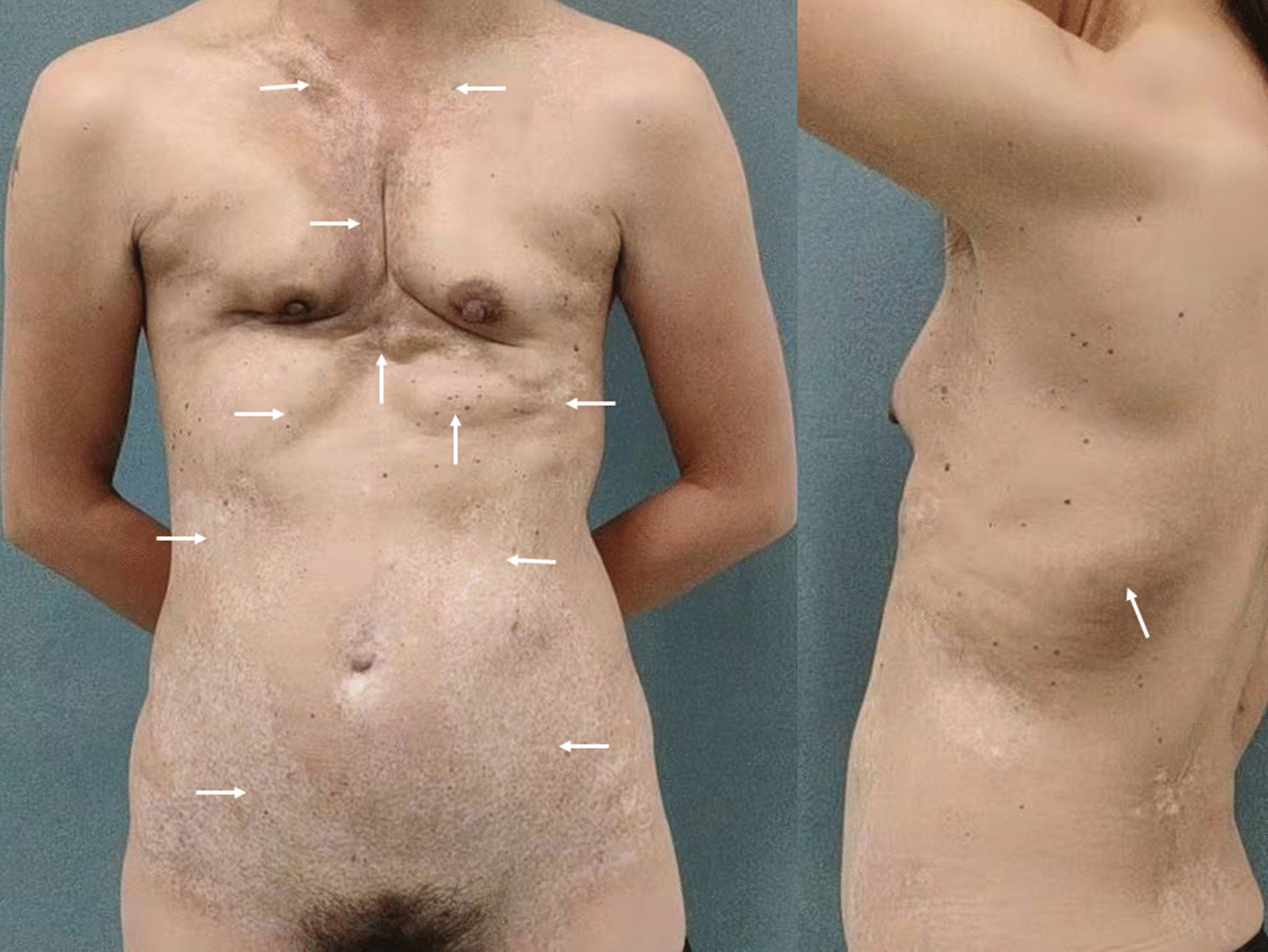
Anterior and left lateral appearance of the patient, and the PAAG migration area (arrow)

During the subsequent four years, she experienced gradual deterioration of breast contracture and chest tightness accompanied with mild breathing difficulties. The patient visited our plastic surgery department seeking a less invasive treatment option. Physical examination revealed malformation of the anterior trunk and hardening of the chest wall. Different lump sizes were detected over the right clavicular region and the bilateral arch of rib. Lumps were also detected in nearly the entire anterior abdomen, excluding the 12 × 14 cm oval area around the navel, and an 11 × 4 cm oval area on the left lower back (Fig. 2).

**Fig.3:**
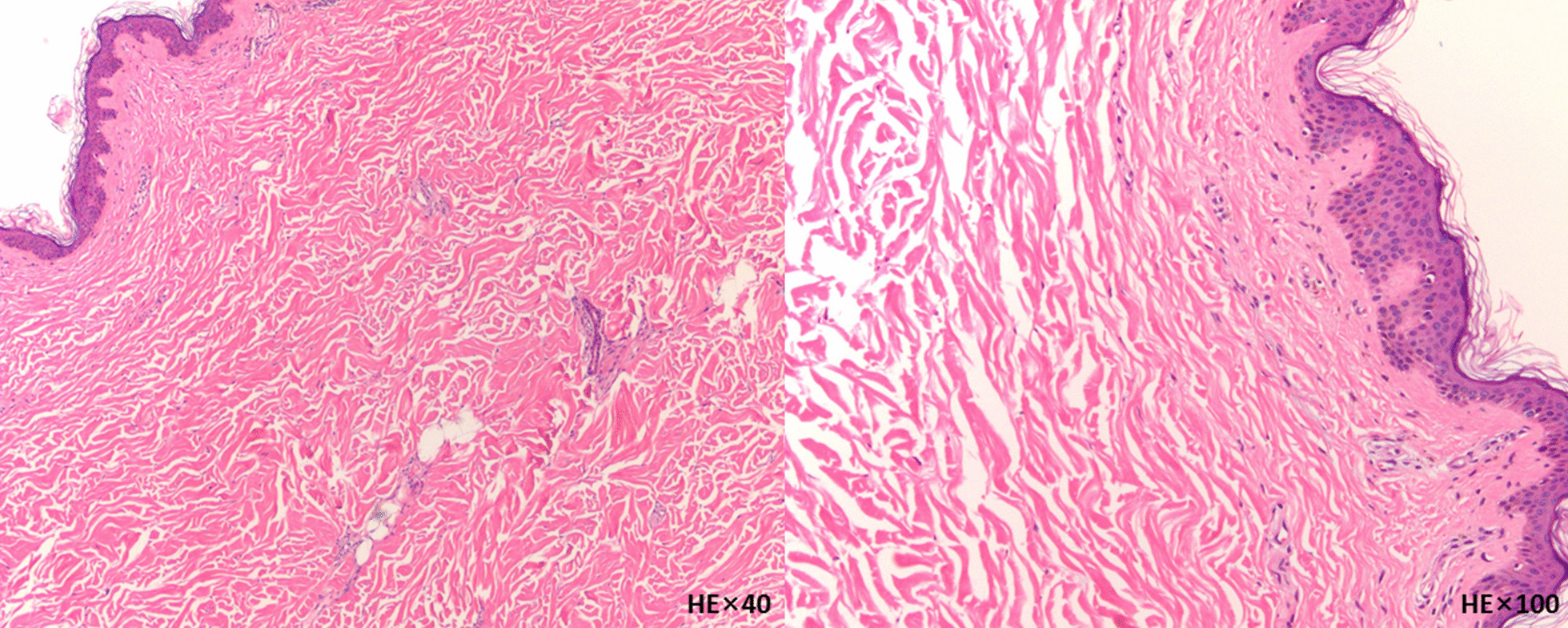
The left image was magnified 40 times and the right one was magnified 100 times. The skin biopsy of breast showed thinner spinous layer, broadened and fused epithelial foot, and lymphocytes infiltration around small vessels in the superficial dermis. Fibrous tissue hyperplasia accompanied by hyalinization occurred in the dermal and skin appendages, and the number of skin appendage was significantly reduced

Pathological examination of the skin showed that the fibrous tissue hyperplasia together with hyalinization in dermal and skin appendages were diminished significantly (Fig. 3). The breast surgical prescription we proposed included removing PAAG, capsule, severe degenerated and harden tissue through multiple incisions (inframammary fold incision and additional incisions above the malposition area of PAAG which cannot be accessed through previous incisions). Incision and partial resection of the contracture tissue would then be performed to release the contracture if necessary. The defect left would be repaired with a free skin or flap grafting. The skin from the removed tissue would be used for grafting. Given the intensity of surgical trauma, we recommended delayed breast reconstruction with autologous tissue. This therapeutic maneuver would still be traumatic, costly and may lead to serious implications. For these reasons, she decided to forego treatment.

## Discussion and conclusion

Polyacrylamide gel (PAAG) is often injected in the retromammary pocket, where it forms a thin capsule. This capsule can break due to gravity, pressure and trauma. Once injected, the hydrogel migrates along the loose connective tissue and extends to the inframammary fold, axilla, sternum, infraclavicular region, abdomen, and perineum [6, 7]. Patients may experience pain, fever, breast deformation which may require clinical treatment. Under the guidance of preoperative MRI images, patients are often operated using the periareola approach and the surgery includes PAAG evacuation, pathologic tissue excision, and pocket irrigation to ensure maximal removing of the PAAG [8]. Breast reconstruction with breast prosthesis is considered a suitable approach for patients with strong desire to undergo breast reconstruction, and with adequate healthy soft tissue to cover the prosthesis.

Herein we report a 49 year-old female patient with familial vitiligo who experienced PAAG migration for nearly 20 years without significant discomfort. Due to the long-term movement, the PAAG was extensively distributed from the clavicular level to the perineum, and from the anterior trunk to the upper back. The skin on the affected area became sclerotic. Tissue degeneration and chronic stimulation of PAAG induced the formation of fibrous tissue and fiber contraction, leading to soft tissue deformation in the anterior trunk and breast contraction. This resulted in chest tightness and labored breathing. The anterior trunk had a larger depigmentation area than the back. Although tissue degeneration and chronic stimulation caused by PAAG has been previously reported [9], this is the first report demonstrating extensive hardening of tissue and breast contracture after long-term PAAG migration without treatment in a patient with vitiligo.

Vitiligo is currently recognized as an autoimmune disease characterized by patches of unpigmented skin. Several autoimmune diseases have been found in patients with vitiligo, such as alopecia areata, Hashimoto's thyroiditis, myasthenia gravis, Graves' disease, Sjogren's syndrome, and systemic lupus erythematosus [10]. Female patients are more likely to develop autoimmune diseases [11, 12].

In this case, tissue hardening occurred in the PAAG migration area where there was higher density of white patches. The condition of the patient was severe due to the long period of PAAG migration. Notably, she developed many complications, such as inflammation, infection, lumps, deformation, displacement, abnormal feeling, systemic toxicity [1, 13], but no PAAG-induced skin contracture or tissue hardening has been reported previously. It is rare that the patient developed long-term PAAG translocation, vitiligo and PAAG-induced skin abnormities simultaneously. Vitiligo might be aggravated by PAAG-induced chronic inflammation. Moreover, the unusual immune response induced by vitiligo may have contributed to the formation of skin sclerosis seen in this case. These hypotheses require further investigation.

The dangers of PAAG gel as a biomaterial filler for breast plastic surgery have been widely recognized and PAAG has been banned in many countries. In the present case, PAAG gel promoted the development of vitiligo and lead to localized sclerosis and contracture, resulting in chest tightness and dyspnea. We hope that more cases and evidences might be found in the future to help to explain the relationship between long-term filler translocation, vitiligo and subcutaneous tissue sclerosis contracture.

## Data Availability

Not applicable.

## Abbreviations

PAAG: Polyacrylamide gel
MRI: Magnetic Resonance Imaging

## References

[1] Cheng NX, Wang YL, Wang JH, Zhang XM, Zhong H (2002). Complications of breast augmentation with injected hydrophilic polyacrylamide gel. Aesthetic Plast Surg.

[2] Unukovych D, Khrapach V, Wickman M, Liljegren A, Mishalov V, Patlazhan G, Sandelin K (2012). Polyacrylamide gel injections for breast augmentation: management of complications in 106 patients, a multicenter study. World J Surg.

[3] Christensen L, Breiting V (2006). Management of postoperative complications of breast augmentation by injected polyacrylamide hydrogel. Aesthetic Plast Surg.

[4] Leung KM, Yeoh GP, Chan KW (2007). Breast pathology in complications associated with polyacrylamide hydrogel (PAAG) mammoplasty. Hong Kong Med J.

[5] State Food and Drug Administration: About prohibited the use of polyacrylamide hydrogel (injection) warning. http://www.sda.gov.cn/WS01/CL0493/93434.html. Accessed 10 May 2015.

[6] Luo SK, Chen GP, Sun ZS, Cheng NX (2011). Our strategy in complication management of augmentation mammaplasty with polyacrylamide hydrogel injection in 235 patients. J Plast Reconstr Aes.

[7] Xiaoling F, Yi C, Zhang Y (2004). Analysis of the complications induced by the polyacrylamide hydrogel injection. Plast Reconstr Surg.

[8] Qian B, Xiong L, Guo K, Wang R, Yang J, Wang Z, Tong J, Sun J (2020). Comprehensive management of breast augmentation with polyacrylamide hydrogel injection based on 15 years of experience: a report on 325 cases. Ann Transl Med.

[9] Qiao Q, Wang X, Sun J, Zhao R, Liu Z, Wang Y, Sun B, Yan Y, Qi K (2005). Management for postoperative complications of breast augmentation by injected polyacrylamide hydrogel. Aesthetic Plast Surg.

[10] Dahir AM, Thomsen SF (2018). Comorbidities in vitiligo: comprehensive review. Int J Dermatol.

[11] Sheth VM, Guo Y, Qureshi AA (2013). Comorbidities associated with vitiligo: a ten-year retrospective study. Dermatology.

[12] Chen YT, Chen YJ, Hwang CY, Lin MW, Chen TJ, Chen CC, Chu SY, Lee DD, Chang YT, Liu HN (2015). Comorbidity profiles in association with vitiligo: a nationwide population-based study in Taiwan. J Eur Acad Dermatol Venereol.

[13] Amin SP, Marmur ES, Goldberg DJ (2004). Complications from injectable polyacrylamide gel, a new nonbiodegradable soft tissue filler. Dermatol Surg.

